# Actinomycosis of the sole of the foot (Madura foot): A rare case report and literature review

**DOI:** 10.1016/j.ijscr.2023.109052

**Published:** 2023-11-13

**Authors:** Boushabi Ayoub, Aitbenali Hicham, Shimi Mohamed

**Affiliations:** Orthopedics and Trauma-surgery Department, MOHAMMED VI University Hospital Center Tangier, Faculty of Medicine and Pharmacy, Abdelmalek Essaidi University, Tangier, Morocco

**Keywords:** Actinomycosis, Foot, Mycetoma, Biopsy

## Abstract

**Introduction and Importance:**

Actinomycosis of the foot is a rare, chronic, granulomatous infectious ailment affecting the skin, dermis, and subcutaneous tissues. It is caused by a fungal or actinomycotic agent, resulting in a pseudotumoral appearance. This pathology typically progresses slowly, leading to delayed diagnosis. The primary objective of this report is to emphasize the rarity and clinical significance of this highly uncommon medical condition.

**Case presentation:**

We present the case of a 45-year-old woman with a mass on the sole of her right foot. The condition was treated through excisional biopsy along with combined antibiotic therapy. Histopathology of the excised mass confirmed the presence of actinomycosis, ultimately confirming the diagnosis. The patient's progress was satisfactory, with no recurrence observed over a 2-year follow-up period.

**Clinical discussion:**

Actinomycosis of the foot is a common pathology in tropical and subtropical regions where climatic conditions promote its emergence. It is a chronic, granulomatous infection of exogenous origin, caused by either fungal (eumycetoma) or bacterial (actinomycetoma) agents. When left unrecognized, these infections can lead to complications, including osteoarticular lesions, with a severe impact on the functional prognosis. The presence of foot swelling should raise suspicion of mycetoma as a diagnosis, with confirmation relying on histological analysis. Management typically involves the aggressive control of invasive soft-tissue masses, often followed by prolonged antibiotic treatment. Skin grafting is a standard method for closing Madura foot defects, and continuous surveillance is necessary due to the potential for actinomycosis recurrence.

**Conclusion:**

In the context of Morocco, actinomycosis of the foot remains sporadic. Identifying these conditions presents significant diagnostic challenges, leading to unfortunate treatment delays. These largely unrecognized conditions have the potential to become more complex by causing osteoarticular damage and lesions, ultimately worsening the functional outlook. Early vigilance is advisable upon observing foot swelling, considering the possibility of actinomycosis of the foot. A definitive diagnosis requires histological analysis, a critical step.

## Highlights

• Actinomycosis of the foot is prevalent in tropical regions with favorable climatic conditions.
• Chronic, granulomatous infection caused by fungal (eumycetoma) or bacterial (actinomycetoma) agents.
• Unrecognized cases may lead to osteoarticular lesions, impacting functional prognosis.
• Suspect mycetoma in the presence of foot swelling; diagnosis confirmation is histological.
• Management involves aggressive control of invasive soft-tissue mass, followed by prolonged antibiotic treatment.

## 1 Introduction

Madura foot, also known as mycetoma, is a regional and persistent granulomatous infection that primarily affects the skin and underlying subcutaneous tissue. This condition is characterized by the growth of parasitic seeds due to the presence of exogenous fungal or actinomycotic etiological agents. Actinomycosis of the foot is a chronic and highly uncommon infectious pathology, with its endemic prevalence primarily noted in tropical countries, although it remains rare in temperate climates.

Actinomycosis of the foot poses a unique challenge in the medical field, primarily due to its rarity, chronic nature, and the difficulty associated with its diagnosis. While this condition remains sporadic in temperate regions, its prevalence in tropical areas, where environmental conditions favor its emergence, underscores its clinical significance. We report a rare case of primary actinomycosis.

## 2 Case history

A 45-year-old female cleaning lady, residing in Tanger, a city in northern Morocco, with no significant previous medical history, presented to our hospital with a history of generalized indurated swelling of the sole of her right foot, dating back approximately 7 months. The patient had sought consultation from several general practitioners and had been prescribed antibiotics, but her condition had not shown any improvement.

**Fig. 1:**
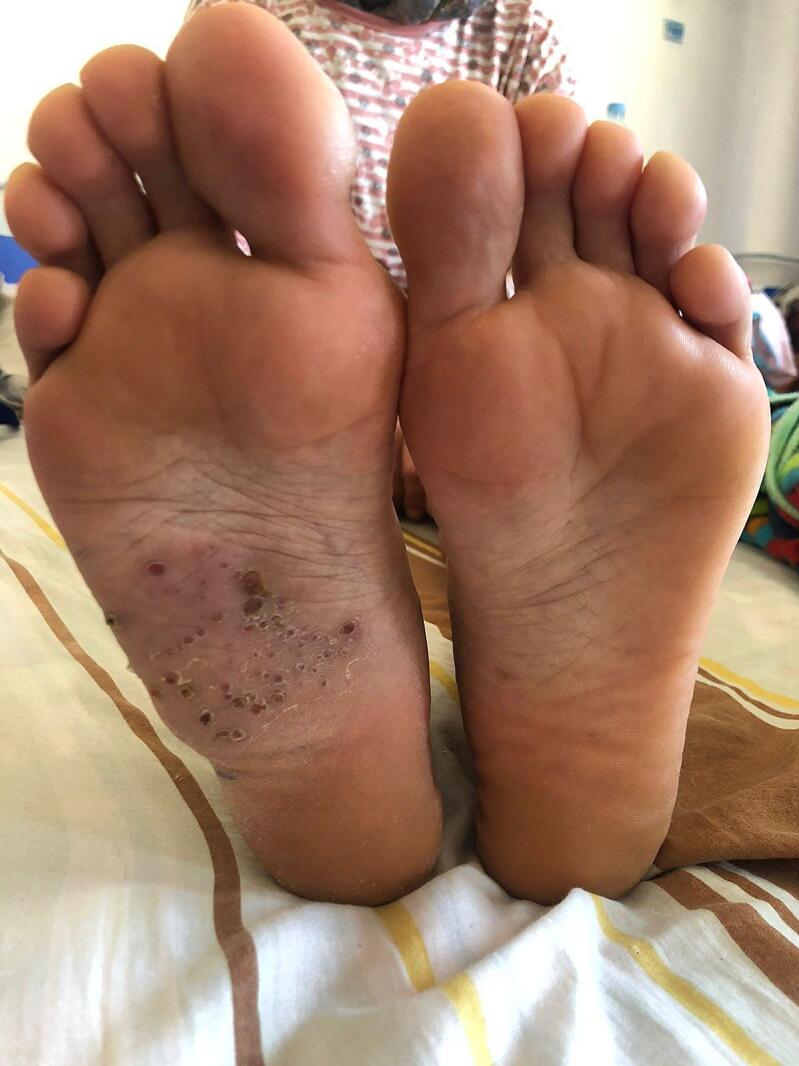
Patient With Actinomycosis Foot Infection With Multiple Sinuses.

The initial lesion began as a single subcutaneous nodule on the sole of her foot, followed by the development of a second nodule two months later. Over time, additional nodules appeared, primarily located on the outer edge and sole of the foot [Fig. 1]. Subsequently, these lesions burst, leading to the formation of sinuses from which yellow granules were intermittently discharged. Over the course of six months, the patient developed a cauliflower-like budding mass measuring 7 cm, which caused significant foot pain, making walking difficult. The mass encompassed the entire sole of her foot and had become sclerotic and hard. Clinical findings at this stage were suggestive of actinomycosis of the foot.

Despite the patient's discomfort and the extensive nature of the mass, she remained afebrile and exhibited no clinical signs of infection such as erythema, warmth, or purulence. Lymphatic striae and palpable inguinal adenopathy were absent. Upon examination, the mass was found to be mildly painful, indurated, infiltrated, and lacked fluctuation. It extended from the plantar surface of the foot, opposite the metatarsals, towards the outer edge of the foot. Small, sporadically draining, crusty lesions with thick, hyperpigmented skin and a few dry fistulae were present on the plantar surface of the mass. However, the mass was devoid of foul odor, drainage areas, and subcutaneous crepitation. Peripheral pulses remained present.

Laboratory results showed a white blood cell count of 9500, mild anemia with a hemoglobin level of 9 g/dL, a platelet count of 250,000, an erythrocyte sedimentation rate of 4 mm/h, and a C-reactive protein level of 2.05 mg/L.

**Fig. 2:**
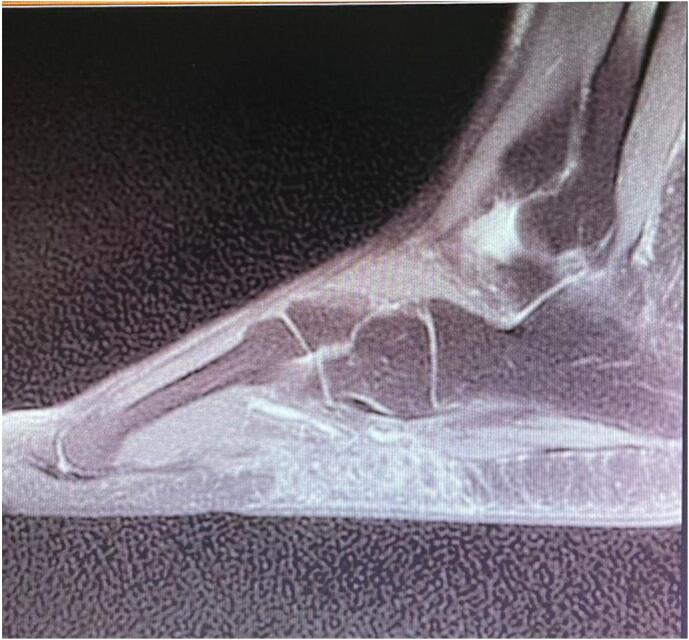
Magnetic resonance imaging showing an extensive inflammatory edematous infiltrate affecting the lateral edge of the foot and the sole of the foot, of dermo-hypodermal location and extending to contact with the plantar fascia without any real visible collection or bone or osteoarticular extension.

Magnetic resonance imaging revealed an extensive inflammatory edematous infiltrate involving the lateral border of the foot and the sole of the foot, located in the dermo-hypodermal region and extending to the contact points with the plantar fascia and the abductor muscle of the fifth toe. No visible collection or osseous or osteoarticular extension was observed [Fig. 2].

**Fig. 3:**
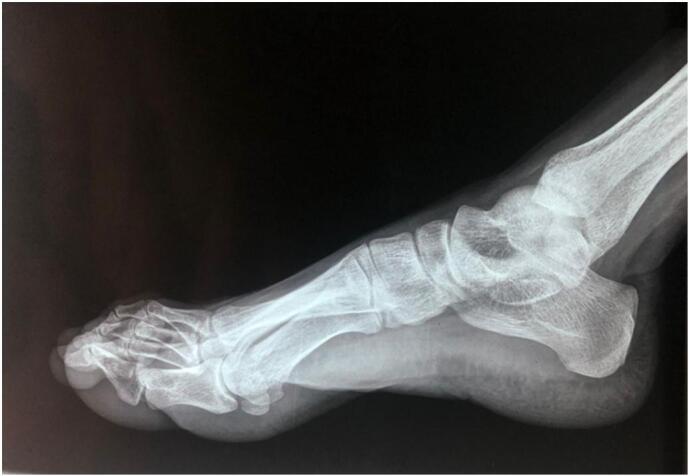
Standard foot X-rays showing soft tissue thickening without visible calcifications, bone lesions or osteolytic images.

Standard radiographs of the foot indicated thickening of the soft tissues, with no calcifications or visible bone lesions or osteolytic images [Fig. 3].

**Fig. 4:**
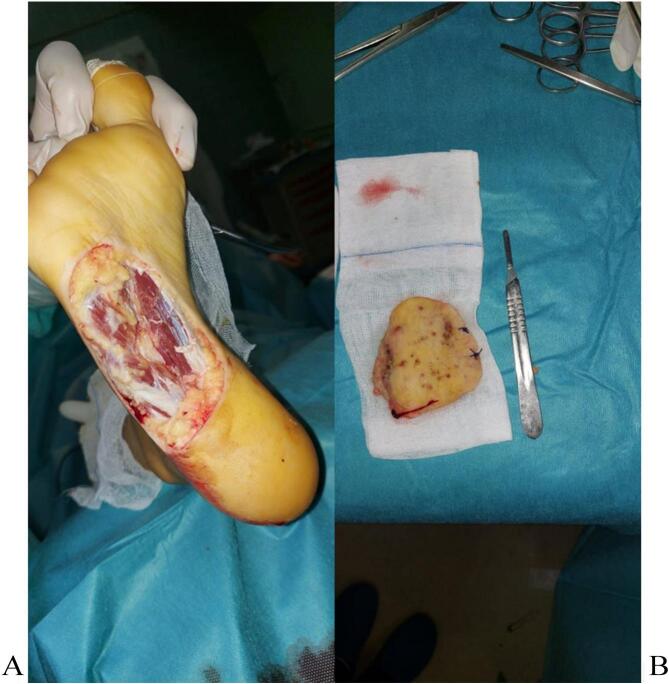
A The surgical approach using an elliptical incision to gain adequate access to the mass B: Excision of the bulk mass.

**Fig. 5:**
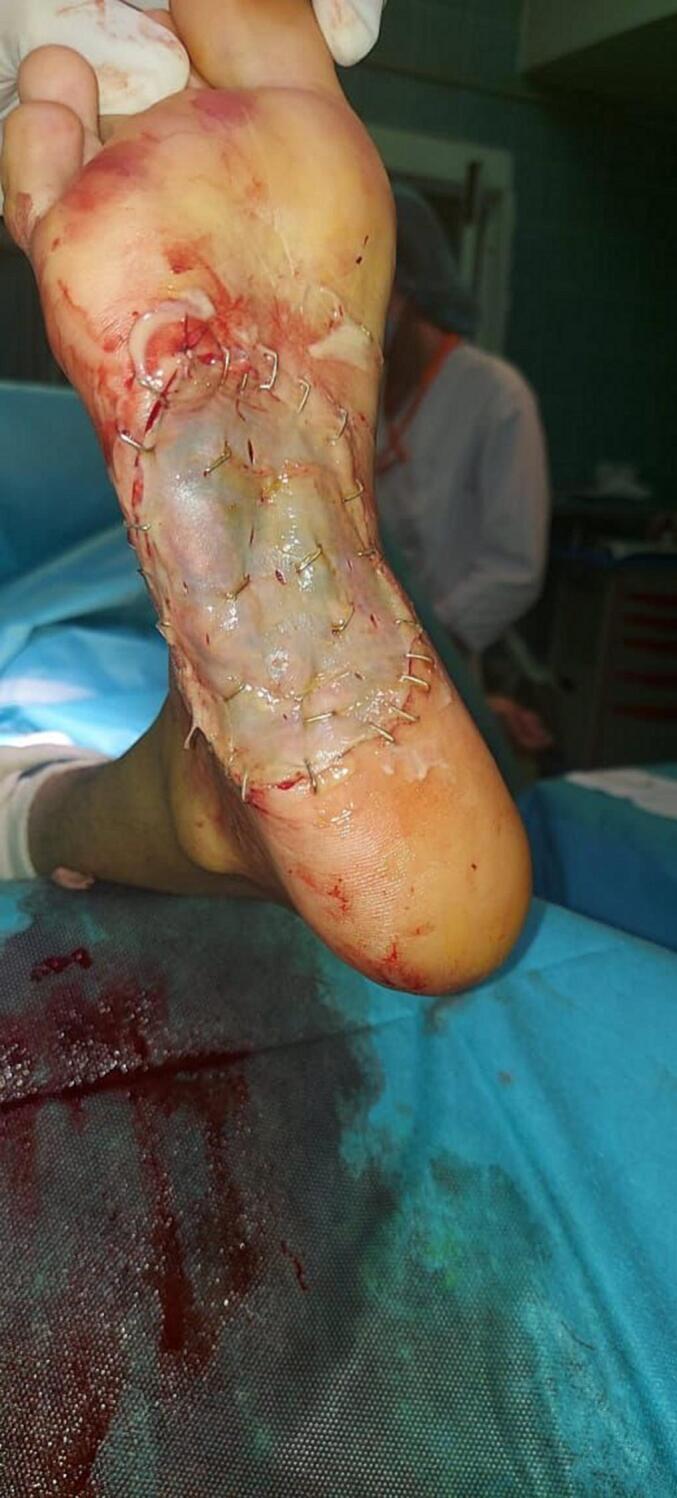
The picture of healed split thickness skin graft.

**Fig. 6:**
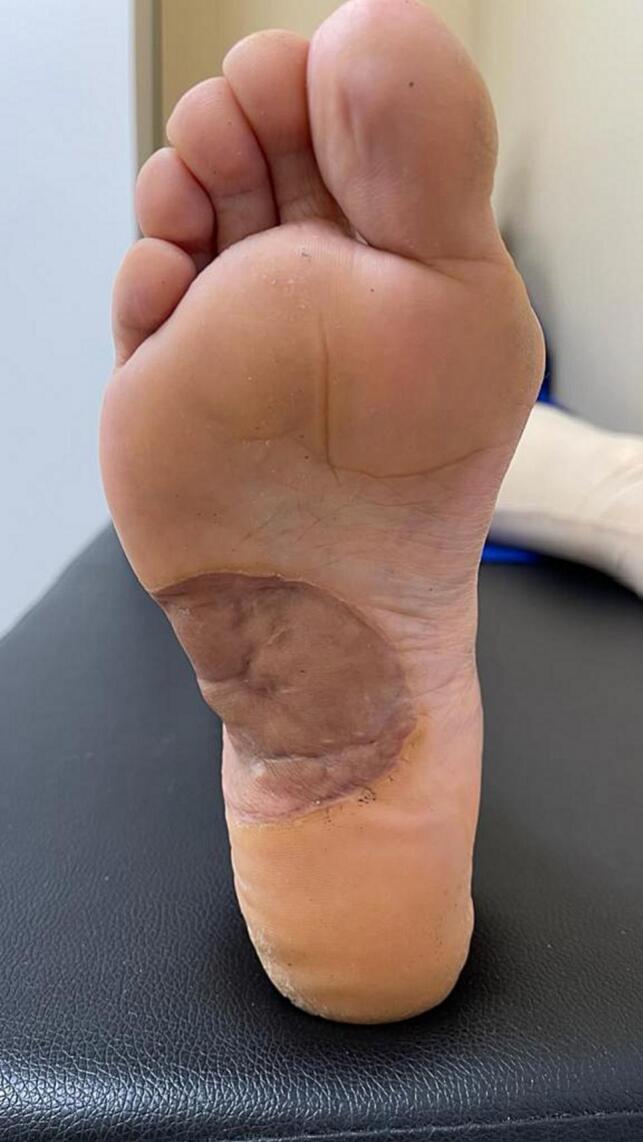
2 years follow-up shows healed suture lines with a reduction in the bulk of the soft tissue mass.

The patient underwent an excisional surgical biopsy, followed by thin skin grafting using the thigh as the donor site [Fig. 4, Fig. 5]. The biopsy confirmed the presence of actinomycotic mycetoma. An antibiotic-sensitive culture of the biopsy was found to be susceptible to penicillin. The patient was initiated on a penicillin G regimen, receiving 24 million units per day for two weeks, followed by cotrimoxazole at a dose of 1600 mg/day for six months. The patient has been under regular follow-up for a period of two years, showing a favorable evolution [Fig. 6].

## 3 Discussion

This section provides an opportunity to delve deeper into the clinical aspects of the presented case and to draw comparisons with previous cases reported in the literature, thus enhancing the clinical context.

Actinomycosis, as demonstrated by this case, remains a formidable diagnostic challenge. While it represents a substantial public health concern in tropical countries, it remains an exceptionally rare pathology in Morocco due to the climate factors at play. Actinomycosis primarily affects soft tissues and the skeletal structure, sometimes leading to severe visceral complications. Madura foot, first described in Madura, India, in 1842, manifests as a chronic, granulomatous infection of exogenous origin, initiated by either fungal (eumycetoma) or bacterial (actinomycetoma) agents [1].

The clinical presentation often commences with traumatic inoculation, facilitated by agents contaminated with soilborne microbes or through skin excoriations. This mechanism of transmission largely explains the frequency of localization in the foot and leg. The incubation period for Madura foot is notably protracted, spanning from a few weeks to several months, or even years [2]. Clinical manifestations usually lead patients to seek consultation during the acute phase.

Typical clinical features include skin fistulas that intermittently open and close, discharging granules of different colors (black granules are indicative of fungal origin, while red granules suggest a bacterial source, and white granules can be fungal or bacterial). Soft tissue swelling is a common manifestation, sometimes accompanied by functional impairment, albeit rarely accompanied by pain. Pain may indicate superinfection or advanced bone involvement. Mycetomas, which develop at the site of inoculation, resemble inflammatory pseudotumors consisting of deep abscesses and skin ulcerations connected through fistulous tracts [3].

The clinical presentation of actinomycosis of the foot presents a significant challenge in terms of differential diagnosis, often involving conditions like tuberculosis, leishmaniasis, and syphilis. Therefore, laboratory data play a pivotal role in confirming the diagnosis. Direct examination of the granules and microbiological analysis on 2 % glucose Sabouraud medium are critical components of the diagnostic process [4].

Microscopic examination of the granules is often the primary means of differentiating between eumycetomas and actinomycotic mycetomas. Fungal grains consist of septate filaments with a diameter exceeding 2 μm, often reaching up to 5 μm. In contrast, actinomycotic grains are consistently less than or equal to 1 μm in diameter [5]. Histological studies, as emphasized by Lee et al. [6], are essential for establishing the diagnosis. Biopsy is especially useful for purely bony forms, as suggested by Ancelle [7].

The invasive nature of the grains results in their progressive infiltration of tissues, affecting fascia, muscles, and bone. In severe cases, this may lead to gangrene. Imaging studies for mycetoma extension focus on assessing bone involvement, with diagnosis relying on imaging modalities. The extent of bone involvement varies based on the duration of the disease, the location of the mycetoma, and the microbe responsible. Characteristic findings include cortical erosions, bone condensation lacunae, periosteal reactions, and marked soft tissue volume increase. Subperiosteal ossifications may also be observed. Magnetic resonance imaging (MRI) serves as a valuable tool for diagnosing the etiology and extent of mycetoma. Mycetomas appear iso- or hyposignal on T1-weighted sequences and slightly hypersignal relative to muscle on T2-weighted sequences. A distinctive feature is the presence of infiltrating masses composed of small compartments separated by fine partitions, containing centrolesional punctiform elements in hyposignal on all sequences, particularly prominent on T2-weighted sequences, creating an almost pathognomonic appearance [4]. Computed tomography (CT) scans do not significantly contribute to the positive diagnosis, as the CT symptomatology of bone involvement mirrors that described in conventional radiology [6,7].

Treatment strategies for actinomycetomas are typically medical-surgical in nature. Medical treatment relies on antibiotics, particularly sulfonamides, when the causative agent is bacterial, and antifungal agents, such as ketoconazole, when the etiology is mycotic. Surgical intervention is necessary from the outset for large lesions, involving extensive resection to preserve the patient's functional potential while minimizing the need for secondary revision [8,9]. Combining medical and surgical treatment is recommended by several authors, with ketoconazole used alongside surgery in cases of fungal mycetomas [10]. Notably, first-line treatment for actinomycetomas typically involves medical management, with few exceptions. Surgical intervention from the outset, especially when dealing with small granules, may inadvertently encourage distant spread. Adoubryn et al. [9] underscore the importance of initiating first-line antibiotic therapy to avoid mutilating surgery and the risk of distant grain metastasis.

It is crucial to understand that clinical resolution of a mycetoma following treatment does not guarantee a definitive cure. Regular clinical follow-up is mandatory for a minimum of three years, as the risk of recurrence significantly decreases only after this period [11,12].

## 4 Conclusion

Actinomycosis of the foot is rare in Morocco but poses significant diagnostic challenges. Early diagnosis and histological confirmation are crucial. This case emphasizes the importance of promptly addressing invasive soft-tissue masses and employing prolonged antibiotic therapy, often complemented by skin grafting. The favorable outcome contributes to our understanding of actinomycosis in Morocco, highlighting the need for vigilance and improved recognition of this condition. Continuing surveillance is essential, given the potential for recurrence. This case report enhances awareness and knowledge of this rare but clinically significant pathology.

## 5 Methods

This work has been reported in line with the SCARE criteria.

## Ethical approval

Not applicable.

## Funding

This research did not receive any specific grant from funding agencies in the public, commercial, or not-for-profit sectors.

## CRediT authorship contribution statement

BOUSHABI Ayoub: study concept, Data collection; data analysis; writing review & editing

AITBENALI Hicham: Contributor, Supervision and data validation

SHIMI Mohammed: supervision and data validation

## Guarantor

BOUSHABI Ayoub

AIT BENALI Hicham

SHIMI Mohammed

## Registration of research studies

As this manuscript was a case report with no new medical device nor surgical techniques, not prior registration is required.

## Consent

Written informed consent was obtained from the patient for publication and any accompanying images. A copy of the written consent is available for review by the Editor-in-Chief of this journal on request.

## Declaration of competing interest

The authors state that they have no conflicts of interest for this report.
